# Patient-Reported Outcomes Measurement Information System as a Clinical Tool for Capturing the Patient Perspective in Pediatric Inflammatory Bowel Disease: A Narrative Review

**DOI:** 10.3390/children11121492

**Published:** 2024-12-06

**Authors:** Sara Azevedo, Ana Isabel Lopes

**Affiliations:** 1 Gastroenterology Unit, Pediatrics Department, Academic Medical Centre of Lisbon, Santa Maria University Hospital—CHULN, Av. Prof. Egas Moniz MB, 1649-028 Lisbon, Portugal; anaisa@ulssm.min-saude.pt; 2 Medical School, University of Lisbon, Avenida Egas Moniz, 1649-028 Lisbon, Portugal

**Keywords:** children, adolescents, Crohn’s disease, health-related quality of life, inflammatory bowel disease, pediatrics, patient-reported outcome measures, PROMIS^®^, pediatric chronic disease responsiveness, self-reporting

## Abstract

Inflammatory bowel disease (IBD) is an immune-mediated chronic disease with a significant impact on quality of life. In pediatric patients, diagnosing and managing IBD is particularly challenging, and IBD often presents as a more severe and progressive disease. Patient-reported outcomes (PROs) are measures of treatment and disease management outcomes reported by patients and/or caregivers. These measures evaluate several aspects of disease management from the patient/caregiver perspective, emphasizing the patient’s real-life experience with the disease and its treatment. PROs represent a model of patient-centered care, facilitating better-informed healthcare decisions. The Patient-Reported Outcomes Measurement Information System (PROMIS) was developed to promote the use of PROs among patients with chronic conditions. Its primary objective is to provide PROs for research and clinical practice throughout the lifespan. The PROMIS is a non-disease-specific instrument for both adults and pediatric patients assessing domains of physical, psychological, and social health, as well as quality of life (QOL). These instruments are designed to be applicable to a wide range of chronic diseases. Despite the initial expectation concerning PROs in assessing pediatric IBD outcomes, objective data in this area have only recently begun to emerge. This narrative review, based on a selection of reliable articles recognized by PubMed and Cochrane Library, aimed to identify and summarize previously published evidence of the usefulness of PROs, particularly the PROMIS, in IBD patients and in the pediatric population. We present an updated perspective, including identification of their general applications and most relevant previous studies, in the mentioned areas and identify knowledge gaps.

## 1. Introduction

Inflammatory bowel disease (IBD) is an immune-mediated chronic disease characterized by gastrointestinal and extra digestive manifestations, with remission and active disease periods. It encompasses Crohn’s disease (CD), ulcerative colitis (UC), and unclassified IBD (IBD-U). In approximately 25% of IBD patients, the diagnosis is established at the pediatric age [1]. Currently, these chronic conditions have no available cure and are widely recognized for their potentially negative impact on quality of life (QOL) [1,2].

Diagnosing IBD in pediatric patients may be quite challenging for both families and patients, as it is often more severe and progressive, with an increased risk of surgery, compared with adult IBD. Additionally, pediatric IBD patients are recognized to have age-specific complications, namely, malnutrition, pubertal delay, linear growth impairment, and decreased bone mineral density [1,3]. In addition to uncomfortable and sometimes disabling symptoms, invasive diagnostic procedures, complex and time-consuming treatment options, and medication side effects, patients and families experience limitations in everyday activities and restrictions in school/professional and social activities [2,4].

Several pediatric studies have documented impaired health-related quality of life (HRQOL) in the physical, psychological, and autonomy domains related to disease activity, requiring specific coping strategies [2,4,5,6,7]. When addressing an IBD patient, a comprehensive approach to function and disability, along with traditional clinical parameters, is desirable. The primary goal of treatment is to achieve and maintain disease control, thereby improving QOL and diminishing the disease burden. A systematic review published in 2014 [8] examined the evidence on HRQOL in pediatric IBD patients and concluded that HRQOL is lower in children and young adults than in adult patients. Understanding pediatric patients’ perspectives requires knowledge of their cognitive and developmental characteristics. Moreover, parents often provide most of the information concerning the child’s health and tend to underestimate the patient’s QOL [7]. Involving the pediatric patient in the management of the disease contributes to a better transition to adult healthcare.

In pediatric IBD, specific QOL instruments such as the IMPACT questionnaire [9,10] have been developed, validated, and used in several studies, both retrospective and prospective, as well as in clinical practice. Furthermore, in the context of pediatric IBD, to address other domains of health and functioning, including mood, sleep, and fatigue disorders, it is often necessary to utilize specific tools in addition to specific IBD tools, increasing the complexity of the assessment process and the respondent burden. Moreover, it is difficult to compare findings across studies in pediatric IBD patients and other pediatric chronic IBD patients.

Generic measures that can be applied to individuals and populations with several health conditions, differentiating groups of overall health and well-being [11], may be useful in IBD patients, as they can capture specific morbidities (such as anxiety and depression) that are not captured by disease-specific measures [12]. Furthermore, they enable comparisons across diverse patient groups and populations with varying health conditions.

## 2. Patient-Reported Outcomes

Patient-reported outcomes (PROs) are health experiences and evaluations assessed by patient reports, including symptoms, assessments of functioning, well-being, health perceptions, and satisfaction with care [13]. They measure the outcomes of treatments and disease management reported by the patient and/or caregivers [4,13,14,15].

The measurement of PROs may be performed via interviews or, more frequently, via written questionnaires delivered through computers or telephone [11]. Initially, developed as an additional outcome tool in clinical trials, their importance was reinforced in 2009 by FDA guidance [16], which advocated for the incorporation of the patient’s perspective in clinical care and research [17].

In the past two decades, PROs have become important tools in healthcare, attracting increasing professional interest as tools for day-to-day management decisions and driving healthcare quality [7,13,14,18,19]. They represent a good model of patient-centered care [13,15,17,20,21].

The use of PROs in a clinical setting is particularly relevant in chronic conditions where patient involvement is central to successful disease management. They provide additional information regarding the burden of the disease or treatment that might be relevant for clinical decisions. PROs assess a person’s feelings, functional status, and well-being and not only traditional clinical outcomes, such as organ system function, disease activity, and comorbidities, allowing a more comprehensive understanding of the impact of chronic disease on patients’ health [22]. As PROs can be monitored without in-office evaluations, they can potentially determine the interval between medical visits [20,21,23,24,25,26]. However, the implementation of PROs in clinical settings faces several barriers, such as time and respondent burden, a lack of time during medical visits, and the absence of standardized measures applicable to different chronic diseases in both research and clinical care [11,27].

In the pediatric setting, PROs are also becoming important endpoints in several chronic conditions [28,29,30,31,32,33,34,35]. In pediatric IBD, different authors have concluded that well-defined, reliable, sensitive, and globally recognized PROs are needed, alongside traditional markers of the disease (endoscopy-based endpoints and/or biomarkers) to facilitate pediatric drug development [18] and clinical practice [36]. Additionally, highlighting the relevance of PROs in IBD, the Selecting Therapeutic Targets in Inflammatory Bowel Disease (STRIDE-II) recommendations [36] have added that the restoration of HRQOL and absence of disability have formal long-term targets, along with endoscopic healing. The STRIDE-II recommends that this target be assessed at an early stage and on a regular basis throughout the disease course, irrespective of other objective markers of inflammation. The authors consider that the development of PROs for use in clinical practice, with high reliability, face validity, construct validity, responsiveness, and feasibility, is an important area of future research, as they should be developed.

An international, cross-disciplinary consensus, including an international working group representing patients, patient associations, gastroenterologists, surgeons, specialist nurses, IBD registries, and PRO measurement methodologists, ref. [37] developed and proposed a standard set of patient-centered outcomes for IBD for patients aged ≥16 years. The suggested outcome domains that are important in adolescent and adult IBD patients include survival and disease control (disease activity/remission, colorectal cancer, anemia), disutility of care (treatment-related complications), healthcare utilization (IBD-related admissions, emergency room visits), and patient-reported outcomes (including quality of life, nutritional status, and impact of fistulae) [37]. Despite the potential impact on care, no PRO measures are yet systematically used in routine clinical practice.

However, several PRO measures have been developed and used in IBD in the past years, from disease-specific tools to generic tools [14]. Many of these PROMs have been used primarily in research and in clinical trials, as requirements to meet the regulatory agencies, and have been formulated from existing measures [14,36]. Table 1 presents the most frequently used PROs used in adult and pediatric IBD. In adult IBD, there are several specific tools that can be employed. In contrast, in pediatric IBD, the available tools are much more limited.

A plethora of IBD-specific PRO measures have been constructed and validated over time, underscoring the complexity and multifaceted nature of the disease’s impact on QOL and highlighting the difficulty in choosing an adequate tool (Table 1). Most PROMs (pediatric and adult) were designed to capture HRLQOL; however, some assess specific aspects of the disease experience, ranging from overall life satisfaction and stress (IBDSI) to detailed concerns about treatment (RFIPC) and the impact on work productivity (WPAI:CD). A minority evaluates PROs regarding disease symptoms (TUMMY-UC and CD-HI).

The diversity in the number of items (ranging from 3 to 71), modes of administration (self-administered vs. interviewer-administered), and response options (from Likert scales to visual analog scales) reflect the complexity of score interpretation and limit everyday use, as the more comprehensive tools have higher respondent-burden. For example, the Crohn’s Disease-Health Index (CD-HI) is notable for its comprehensive nature, comprising 71 items, which allows for a detailed assessment of various health domains. In contrast, shorter forms, such as the SIBDQ, provide a more streamlined evaluation, which is suitable for quicker assessments.

The Crohn’s Disease Health Index (CD-HI) has recently been developed and validated as a disease-specific PROM with the aim of measuring the symptomatic burden of CD in adults [52]. Although the research results supported the use of the CD-HI as a valid, sensitive, reliable, and relevant PRO in CD [52], its use is not yet generalized. It was only tested in adult CD patients, and thus is not applicable to pediatric CD patients.

Language availability is also a crucial consideration, as multilanguage translation and validation is fundamental to accommodate diverse patient populations. Finally, the scarcity of pediatric PROMs underscores the necessity of age-appropriate assessments, acknowledging that IBD’s impact can differ significantly between these groups and emphasizing the importance of developing and validating age-specific tools.

The multitude of IBD-specific PRO measures suggest the necessity for continued refinement and standardization to ensure comprehensive, reliable, and universally applicable assessment tools, thus suggesting the usefulness of more generic PRO measures. This is further reinforced by the STRIDE II initiative. It recommends that QOL (including food-related QOL), disability, fatigue, depression, anxiety, sexual dysfunction, and body image should be considered important PROMs that must be regularly assessed in patients with IBD (both adult and pediatric) [36].

Current generic PRO measuring tools were developed as research tools and may not be appropriate to routine clinical practice; shorter and simpler measuring tools for everyday use are of the utmost importance. Also to consider, in a clinical context, the use of a generic instrument [11,14,23] may better assess domains of general function, well-being, or quality of life; furthermore, condition-specific PROs may better measure a specific symptom or set of symptoms directly related to a condition or a condition-specific intervention [11].

In both generic and specific measures, there are advantages and disadvantages to consider. Generic PROMs can be applied to individuals and populations with health conditions. They can differentiate groups on indexes of overall health and well-being. They allow for comparability across patients and populations with different conditions. They facilitate the score interpretation, as they usually produce normative data and have a reference population. This enables comparison with information about various disease conditions. However, generic PROMs may be less responsive to focal changes, with the potential for underestimating health changes in specific patient populations. Additionally, they may fail to capture important condition-specific concerns. Furthermore, they are more suitable for comparison across groups than for individual use [11]. Also, useful generic PROs for clinical practice must have high reliability, face validity, construct validity, responsiveness, and feasibility [36].

## 3. Patient-Reported Outcome Measurement Information System (PROMIS)

In 2004, the National Institutes of Health (NIH) from the United States launched the Patient-Reported Outcome Measurement Information System (PROMIS), which was developed to address, investigate, and promote the use of PROs among patients with chronic conditions and to provide the next generation of standardized PRO measures in pediatric and adult health with improved reliability and validity [55,56,57]. Their key objective is to provide PROs for research and clinical practice across the life course.

The PROMIS is a set of nondisease-specific instruments, one for adults and one for pediatric patients, that assess domains of physical, psychological, and social health, as well as QOL. These instruments are designed to be applicable to a range of chronic diseases, offering advantages over disease-specific instruments, as they enable comparisons among different domains of health and across a comprehensive range of chronic diseases affecting both adults and children [19,28,29,30,32,33,34,35,58,59].

The PROMIS has constructed and validated item banks that can be used to assess a range of symptoms and health concepts. These item banks enable the efficient and interpretable clinical trial and clinical practice applications of PROs [55]. An item bank is a repository of meticulously calibrated questions that delineate and quantify a universal concept, thereby providing a functional definition of a trait [55]. PROMIS item banks were developed to reflect a patient’s conceptualization on important symptoms and functions in one’s day-to-day life. PROMIS item banks are organized in domains is a set of non-disease-specific questionnaires, evaluating physical, psychological, and social health as well as QOL domains.

PROMIS measures have different methods of administration, including paper-and-pencil tests and computer-based administration. Paper-based PROMIS measures are available as “respondent ready” PDFs. Paper-based measures have different types regarding length, including the fixed length short form, consisting of a fixed set of four to ten items or questions for each domain, minimizing respondent burden and enhance long-term cohort retention [19,29,33,35].

**Figure 1:**
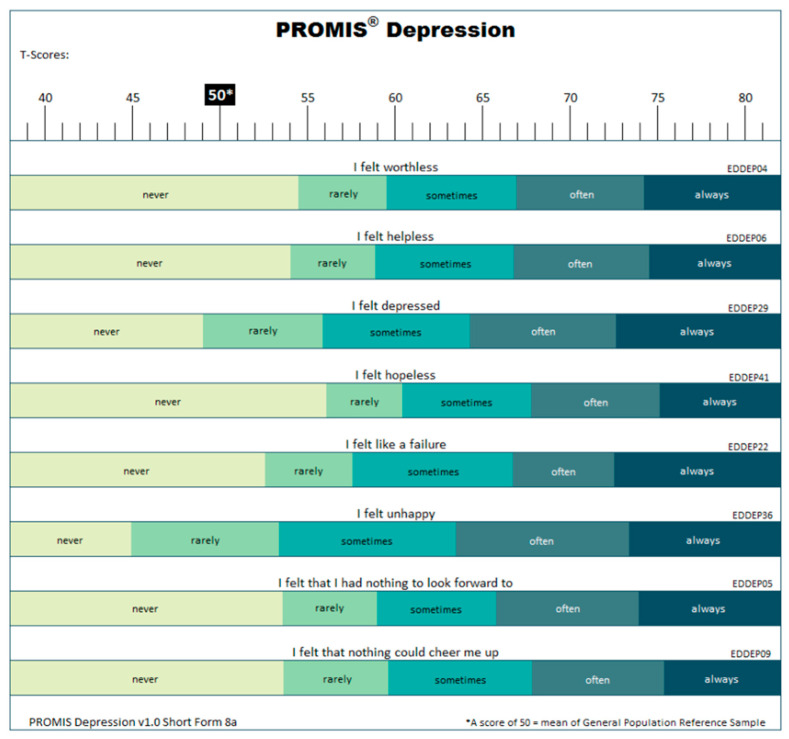
“T score maps” (from www.nihpromis.org).

The PROMIS instruments are calibrated via a T score metric, with the mean of the original calibration population equal to 50 and the standard deviation (SD) equal to 10. Higher scores in any PROMIS domain indicate that more of the domain is being measured; for example, higher scores for anxiety, depression, fatigue, and pain interference indicate poorer well-being, whereas higher scores for peer relationships indicate better well-being [60]. Figure 1 illustrates the measurement of the domains.

The ability of a PRO measure to detect change over time, when expected, defines responsiveness and supports the efficacy of the intervention or the disease treatment impact.

The term minimally important difference (MID) is used to describe the smallest differences in PRO scores that can be detected as a clinically meaningful change in the outcome that the PRO is designed to measure [61,62]. Any discrepancies in PRO scores that are less than the MID are likely attributable to measurement error rather than an authentic alteration in the outcome. Studies in adults suggest that MIDs for many PROMIS items range from 2 to 6 [61]. MIDs are an objective measure of assessing responsiveness over time.

## 4. Pediatric PROMIS

Like the adult PROMIS project, the PROMIS pediatric multisite initiative developed, over the past ten years, including several pediatric self-report item banks for individuals aged 8–17 years across five general health domains, namely, physical function, pain, fatigue, emotional health, and social health, on the basis of the larger PROMIS network, creating pediatric self-report scales measuring unidimensional health attributes (domains) of depressive symptoms, anxiety, anger, pain interference, peer relationships, fatigue, and physical functioning [60,63,64].

PROMIS pediatric measures were developed, using the same methodology as PROMIS adult measures, via qualitative and quantitative methods, including focus groups, expert item reviews, cognitive interviews, and item administration to a large population of North American children and adolescents. These methods created item banks specific to selected domains [60,64]. Similar to the publicly available, cost-free adult PROMIS measures, the pediatric measures offer flexible modes of administration, provide parent–proxy report scales for children aged 5–7 years, can be customized to the disorder’s distinctive characteristics, have the potential to link measures across pediatric and adult age groups [65], and are calibrated via the same methodology as adult PROMIS instruments. Despite the lack of established MIDs in PROMIS pediatric measures, recent research employing adolescent patients, parents, and physicians as judges of clinically important differences in scores indicates MIDs of 2–3 for multiple pediatric PROMIS instruments [61]. A recent published study highlighted another possible approach, the use of disease-specific MIDs [62]. The authors stated that some of the samples utilized to establish the metric and to determine the mean and standard deviation of the distributions were not representative of the general population, nor were they representative of a clinical population. Thus, they suggest the possibility of establishing disease-specific percentiles for PROMIS pediatric measures and estimating clinically important changes by percentile changes (approximately one-half standard deviation units) [62].

Data from several studies reinforce that pediatric PROMIS measures are efficient, precise, and valid across various diseases for assessing patient-reported symptoms and quality of life. They have been studied in chronic diseases, including sickle cell disease [30,33], kidney disease [31,33], cancer [35,66], rehabilitative needs [28,58], obesity [33], asthma [33,34], rheumatic disease [33,34], and IBD [22,32,62,67,68]. These cross-sectional studies validated the PROMIS pediatric measures and demonstrated good discrimination among groups in terms of disease activity and severity, emphasizing the functional burden of chronic conditions and the importance of integrating self-reported PROs in the clinical assessment [22]. Furthermore, research, regarding pediatric PROMIS measures, demonstrated evidence for the responsiveness of PROMIS pediatric measures to clinical disease states in several chronic pediatric illnesses [12,30,34,68,69]. PROMIS pediatric measures have discriminative ability among different clinically meaningful subgroups within several chronic illnesses and allow comparisons of results across different chronic diseases [30], thus supporting the utilization of PROMIS pediatric measures in clinical and research context.

All studies, however, emphasize the necessity of future research, including a broader pediatric population (in number and with diverse demographic characteristics), as well as the incorporation of biological and/or objective clinical anchors of disease.

## 5. IBD and PROMIS

The management of adult and pediatric CD patients has advanced significantly, with evolving therapeutic options and ambitious treatment goals. There is a paradigm shift from a disease-centric approach to a patient-centric approach, as emphasized by the STRIDE II [36] recommendations for treat-to-target strategies in IBD.

In adult IBD patients, the use of the PROMIS was first reported in a cross-sectional and longitudinal study [19] aiming to analyze and evaluate associations between PROMIS measures and validated disease activity indices (Short Crohn’s Disease Activity Index and Simple Clinical Colitis Activity Index) and the Short IBD Questionnaire quality of life instrument. The measured PROMIS domains included anxiety, depression, fatigue, sleep disturbance, satisfaction with social role, and pain interference. The study concluded that disease control was related to several health outcome measures reported by patients, providing strong support for the construct validity of the PROMIS in the IBD population. This study included a large number of geographically diverse patients; however, it was not performed in a real clinical setting, the IBD type and activity were self-reported, and clinical anchor disease assessments were lacking. Despite these limitations, this study represents a significant advancement in the applicability of the PROMIS in IBD patients.

Other studies sought to validate different PROMIS patient-reported measures in IBD patients [70,71,72]. These studies evaluated social and emotional functioning [71,72], and sexual function [72] in adult IBD patients, supporting the validity of PROMIS measures in assessing HRQOL and functioning and further supporting the potential interest and relevance of using PROMIS tools in the clinical setting.

Recent studies addressing the PROMIS in IBD patients have extended the measures applied to evaluate both gastrointestinal symptoms [70] and the burden of disease in different areas [12,73], enhancing its validity, reliability, and responsiveness for assessing general HRQOL and treatment response in IBD clinical trials and in the clinical setting.

Data on PROs in pediatric IBD are emerging [22,32,62,68,69]. These studies, which use several PROMIS pediatric measures, consistently document similar results to adult IBD data, enhancing reliability with good correlation with clinical disease activity scores and with pediatric IBD-specific HRQOL scales.

In this setting, PROMIS pediatric tools have been used for the first time in a web-based cohort of 276 North American children with CD276 (aged 9–17 years) and in their parents [32]. Data on self-reported CD activity, HRQOL (IMPACT-III), and the PROMIS domains of pain interference, anxiety, depression, fatigue, and peer relationships were recorded at baseline and six months later. Furthermore, children with improved Crohn’s disease activity reported improved scores on all PROMIS pediatric measures, and children with worsened Crohn’s disease activity reported worse scores for all domains except anxiety. This study revealed that PROMIS scores were significantly related to CD activity in a linear and clinically meaningful manner and reflected the change in CD activity over a 6-month period, supporting the validity and responsiveness of the pediatric PROMIS within this time period.

More recently, two longitudinal studies documented evidence for the longitudinal responsiveness of the PROMIS pediatric measures to changes in disease status and HRQOL in pediatric IBD patients and in both CD and UC pediatric patients [68,69]. Both studies were cross-sectional and longitudinal (follow-up of 6 months) and recruited patients aged 9–17 years from a North American internet-based cohort of children with IBD (IBD Partners Kids & Teens). Disease activity scores (short Crohn’s Disease Activity Index and Pediatric Ulcerative Colitis Activity Index), the IMPACT-III, and 5 PROMIS pediatric measures (anxiety, depressive symptoms, pain interference, fatigue, and peer relationships) were compared to examine the extent to which the PROMIS pediatric measures responded to changes in clinical scores and IMPACT-III scores. A total of 635 pediatric IBD patients were included (544 CD patients and 91 UC patients), and in both studies, the results were similar, documenting a change in all PROMIS pediatric domains related to changes in clinical scores and changes in IMPACT-III scores. In both the cross-sectional and longitudinal analyses, better PROMIS pediatric scores were associated with lower disease activity and improved IMPACT-III scores. These studies support the use of the PROMIS in pediatric IBD patients in clinical research as well as in patient care.

## 6. Conclusions

The incorporation of PROs into clinical practice for pediatric inflammatory bowel disease (IBD) represents a significant advancement in the delivery of patient-centered care. The incorporation of PROs into the management of chronic conditions such as IBD is particularly advantageous, as it allows for a more comprehensive understanding of the patient’s health status, encompassing not only clinical symptoms but also the physical, psychological, and social dimensions of health. This holistic approach aligns treatment plans more closely with patient needs, thereby fostering improved adherence, satisfaction, and overall health outcomes. Conventional clinical assessments frequently prove inadequate for capturing the comprehensive impact of IBD on daily life. In contrast, PROs offer a valuable means of addressing this gap by providing crucial insights into emotional well-being, social functioning, and QOL. Furthermore, they facilitate enhanced communication between patients, caregivers, and healthcare providers, thereby enabling more informed and effective decision-making.

The advances of technology, including electronic health records and automated systems, has significantly reduced the burden associated with implementing PROs, thereby facilitating their routine integration. Among the available tools, the PROMIS is distinguished by its validity, reliability, and responsiveness in assessing QOL and health outcomes in pediatric IBD.

The PROMIS represents a pivotal tool for the assessment of QOL and health outcomes in pediatric IBD. It addresses the key limitations of traditional disease-specific tools by providing a standardized, multidimensional framework capable of evaluating physical, emotional, and social health across diverse populations. The validity, reliability, and responsiveness of PROMIS pediatric measures have been demonstrated by emerging evidence, which also shows strong correlations with clinical disease activity and pediatric-specific QOL scales like IMPACT-III. The PROMIS has demonstrated sensitivity to changes in disease status and treatment response, underscoring its utility in both research and routine clinical care. Furthermore, its efficiency, minimal respondent burden, and adaptability for various chronic conditions enhance its clinical relevance. However, challenges remain, including the need for broader demographic validation and integration with objective clinical markers. Ultimately, the PROMIS has the potential to advance patient-centered care and improve outcomes for pediatric IBD patients.

In conclusion, the incorporation of PROs, particularly through instruments like the PROMIS, is not merely an additional component of care but is, in fact, a fundamental element of contemporary pediatric IBD management. By prioritizing the patient’s voice, healthcare providers can deliver personalized care that significantly enhances quality of life, well-being, and long-term outcomes for children with IBD.

## 7. Future Perspectives

Notwithstanding advancements, challenges persist in integrating the PROMIS into pediatric IBD care. The lack of established MIDs and the necessity for broader validation across diverse IBD populations underscore the significance of identifying key research priorities. Practical impediments, such as time constraints and respondent burden, must be addressed through the implementation of strategies that leverage technology to enhance the administration, collection, and engagement of patients in the process of PRO administration.

Future research should concentrate on defining the optimal set of PROMIS measures for IBD management. These should encompass a comprehensive range of health and quality of life outcomes that extend beyond specific disease-related symptoms. They should be applicable to both CD and ulcerative colitis UC patients, as well as both adult and pediatric patients, including proxy measures. Furthermore, they should be feasible for application in routine clinical practice, responsive to change, and meaningful to pediatric patients [37].

Longitudinal and real-world studies are essential to validate the responsiveness of PROMIS measures at different disease stages. Aligning these efforts with the STRIDE-II recommendations will promote a more holistic, patient-centered approach to IBD care.

## Figures and Tables

**Table 1:** IBD-specific PRO measures used in adult and pediatric IBD, adapted from [2,38,39].

PRO Measure	Outcome Measured	Items n	Mode of Administration	Response Options	Languages
Adult					
Inflammatory Bowel Disease Stress Index (IBDSI) [40]	Quality of life (Overall life satisfaction, worries about health, psychosomatic symptomatology relationships, sexuality, body image, recreation)	8	Self, Interviewer	Rating 0 (=no impact)–3 (=great impact)	English
Inflammatory Bowel Disease Questionnaire (IBDQ-32) [41]	Quality of life	25	Self, Interviewer	7-point Likert	Multiple
Inflammatory Bowel Disease Quality of Life Questionnaire (IBDQOL) [42]	Quality of life	36	Self	7-point responses	Multiple
Inflammatory Bowel Disease Questionnaire—short form (IBDQ-9) [43]	Quality of life	9	Self, Interviewer	1–7 Likert	English
Short Inflammatory Bowel Disease Questionnaire (SIBDQ) [44]	Quality of life	10	Self, Interviewer	1–7 Likert	Multiple
The Padova Inflammatory Bowel Disease Quality of Life (PIBDQL) [45]	Quality of life	29	Self	0–3 Likert	Italian
The Short Health Scale for Ulcerative Colitis [46]	Quality of life	4	Self	Visual analog scale	Swedish
The Edinburgh Inflammatory Bowel Disease Questionnaire [47]	Quality of life	13	Self	0–1 or 0–3 Likert	English
The UK Crohn’s and ulcerative colitis questionnaire [48]	Quality of life	8	Self	0–3 Likert	English
Cleveland Global Quality of life (Faszio Score) (CGQL) [49]	Quality of life after pouch surgery	3	Self, Interviewer	Rating 0–10	English
Rating Form of IBD Patient Concerns (RFIPC) [50]	Concerns associated with IBD and treatments	35	Self	Visual analog scale	Multiple
Work Productivity and Activity Impairment: Crohn’s Disease (WPAI:CD) [51]	Work and activity impairment	6	Self	0–10 number of hours	Multiple
Crohn’s Disease–Health Index (CD-HI) [52]	Fatigue, dietary restrictions, gastrointestinal health, sleep and daytime sleepiness, bowel control, emotional health, joint health, pain, neck and back health, activity, participation social health, skin health	71	Self	Rating 0–100 (100 = maximum disease burden)	English
Pediatric					
IMPACT III [53]	Quality of life	35	Self	0–5 Likert	Multiple
TUMMY-UC [54]	Symptoms for pediatric ulcerative colitis	8	Self	Rating 0 (= better)–60 (= worst)	English

## Data Availability

Data are contained within the article.

## Abbreviations

Crohn’s disease (CD), health-related quality of life (HRQOL), inflammatory bowel disease (IBD), patient-reported outcomes (PROs), patient-reported outcome measurement information system (PROMIS), quality of life (QOL), therapeutic targets in inflammatory bowel disease (STRIDE-II), ulcerative colitis (UC), unclassified inflammatory bowel disease (IBD-U).
